# Added Value of Deep Sequencing Relative to Population Sequencing in Heavily Pre-Treated HIV-1-Infected Subjects

**DOI:** 10.1371/journal.pone.0019461

**Published:** 2011-05-13

**Authors:** Francisco M. Codoñer, Christian Pou, Alexander Thielen, Federico García, Rafael Delgado, David Dalmau, Miguel Álvarez-Tejado, Lidia Ruiz, Bonaventura Clotet, Roger Paredes

**Affiliations:** 1 Institut de Recerca de la SIDA irsiCaixa-HIVACAT, Badalona, Spain; 2 Unitat VIH, Hospital Universitari Germans Trias I Pujol, Badalona, Spain; 3 Max Planck Institute für Informatik, Saarbücken, Germany; 4 Hospital San Cecilio, Granada, Spain; 5 Hospital 12 de Octubre, Madrid, Spain; 6 Hospital Universitari Mutua Terrassa, Terrassa, Spain; 7 Roche Diagnostics SL, Sant Cugat del Vallès, Spain; University of Sao Paulo, Brazil

## Abstract

**Objective:**

To explore the potential of deep HIV-1 sequencing for adding clinically relevant information relative to viral population sequencing in heavily pre-treated HIV-1-infected subjects.

**Methods:**

In a proof-of-concept study, deep sequencing was compared to population sequencing in HIV-1-infected individuals with previous triple-class virological failure who also developed virologic failure to deep salvage therapy including, at least, darunavir, tipranavir, etravirine or raltegravir. Viral susceptibility was inferred before salvage therapy initiation and at virological failure using deep and population sequencing genotypes interpreted with the HIVdb, Rega and ANRS algorithms. The threshold level for mutant detection with deep sequencing was 1%.

**Results:**

7 subjects with previous exposure to a median of 15 antiretrovirals during a median of 13 years were included. Deep salvage therapy included darunavir, tipranavir, etravirine or raltegravir in 4, 2, 2 and 5 subjects, respectively. Self-reported treatment adherence was adequate in 4 and partial in 2; one individual underwent treatment interruption during follow-up. Deep sequencing detected all mutations found by population sequencing and identified additional resistance mutations in all but one individual, predominantly after virological failure to deep salvage therapy. Additional genotypic information led to consistent decreases in predicted susceptibility to etravirine, efavirenz, nucleoside reverse transcriptase inhibitors and indinavir in 2, 1, 2 and 1 subject, respectively. Deep sequencing data did not consistently modify the susceptibility predictions achieved with population sequencing for darunavir, tipranavir or raltegravir.

**Conclusions:**

In this subset of heavily pre-treated individuals, deep sequencing improved the assessment of genotypic resistance to etravirine, but did not consistently provide additional information on darunavir, tipranavir or raltegravir susceptibility. These data may inform the design of future studies addressing the clinical value of minority drug-resistant variants in treatment-experienced subjects.

## Introduction

The rate of virological failure of the 3 original drug classes is low, but not negligible, and does not appear to diminish over time from starting antiretroviral therapy.[1] If this trend continues, many patients will require newer drugs to maintain viral suppression and accurate resistance tests will be needed to guide clinical management. Deep HIV-1 sequencing (454 Life Sciences/Roche Diagnostics) could potentially improve genotypic resistance assessments because it detects the same range of mutants than Sanger viral population sequencing, but with higher sensitivity [2].

Studies have shown that pre-existing minority drug-resistant mutants increase the risk of virological failure to first-line antiretroviral therapy with non-nucleoside reverse transcriptase inhibitors (NNRTIs) [3], [4], [5], [6], [7]. Conversely, low-frequency drug-resistant variants do not affect virological outcomes of first-line therapy including drugs with high genetic barrier, like ritonavir-boosted protease inhibitors (PIr) [8]. Whereas most studies addressing the role of minority variants have been performed in antiretroviral-naïve subjects [2], [3], [4], [5], [6], [7], [9], [10], [11], [12], [13], less information exists on the clinical significance of minority mutants in antiretroviral-experienced individuals. [14], [15], [16]

It is particularly uncertain if ultrasensitive genotypic tests could provide clinically relevant information in heavily pre-treated HIV-1-infected subjects beyond that provided by standard population sequencing genotypic tests. On one hand, detection of additional minority drug-resistant mutants could improve the assessment of viral susceptibility to drugs with intermediate or high drug genetic barrier. On the other hand, the presence of extensive drug resistance could compromise the ability of deep sequencing to add relevant genotypic information to that already obtained with population sequencing, particularly when alternative treatment options are severely limited. Moreover, mutant fixation during virological failure in the presence of ART pressure could potentially complicate the detection of additional low-frequency variants [16].

We therefore sought to explore the potential of deep sequencing to provide additional, clinically relevant genotypic information that could be used to improve treatment decisions in heavily pre-treated HIV-1-infected subjects, relative to population sequencing.

## Methods

### Subjects

This proof-of-concept observational study included HIV-1-infected adults with previous virological failure (VF) to protease inhibitors (PIs), nucleos(t)ide (NRTIs) and non-nucleoside reverse transcriptase inhibitors (NNRTIs), who developed virological failure to deep salvage therapy including, at least, darunavir, tipranavir, etravirine or raltegravir. Virological failure was defined as the presence of HIV-1 RNA levels >200 copies/mL 24 weeks after salvage therapy initiation or beyond. Adherence was self-reported by the patient and collected from medical charts. Adequate adherence was defined as intake of all medication doses. Partial adherence was defined as the presence of missed doses during treatment. Treatment interruption meant the complete interruption of therapy during follow-up. The Institutional Review Board of the Hospital Universitari Germans Trias i Pujol, Badalona, Spain, approved the study; participants provided written informed consent for retrospective sample testing.

### HIV RNA extraction and reverse transcription

HIV-1 RNA was extracted from 1 mL of plasma within 4 weeks before initiation of deep salvage therapy (baseline) and at virological failure (QIAamp UltraSens Virus Kit^TM^, QIAGEN, Valencia, CA). Three One-Step RT-PCRs (SuperScript® III One-Step RT-PCR System with Platinum® Taq High Fidelity, Invitrogen, Carlsbad, CA) were performed in parallel per each sample. Primers used were 1571-L23 (HXB2: 1417→1440) 5′-ATT TCT CCT ACT GGG ATA GGT GG-3′ and 5464-L27 (HXB2: 5464→5438) 5′-CCT TGT TAT GTC CTG CTT GAT ATT CAC-3′. Cycling conditions were: 30 min. at 52°C, 2 min. at 94°C; 30 sec. at 94°C, 30 sec. at 56°C, 4 min. at 68°C, for 20 cycles; followed by 5 min. at 68°C. Triplicate RT-PCR products were pooled, column-purified (QIAquick PCR Purification Kit, QIAGEN, Valencia, CA) and used for both viral population and deep HIV-1 sequencing.

### Viral population genotyping

Triplicate nested PCRs were performed in parallel (Platinum® Taq DNA Polymerase High Fidelity, Invitrogen, Carlsbad, CA) with primers 2084-U26 (HXB2: 2084→2109) 5′-ATT TTT TAG GGA AGA TCT GGC CTT CC-3′ and 5456-L26 (HXB2: 5456→5431) 5′-TGT CCT GCT TGA TAT TCA CAC CTA GG-3′. Cycling conditions were: 2 min. at 94°C; 30 sec. at 94°C, 30 sec. at 56°C, 4 min. at 68°C, for 20 cycles; followed by 5 min. at 68°C. Nested PCR products were pooled and column-purified. Protease (PR), reverse transcriptase (RT) and integrase (IN) were sequenced in-house (BigDye v3.1, Applied Biosystems, Foster City, CA, USA) and resolved by capillary electrophoresis (ABI 7000, Foster City, CA, USA).

### Deep HIV-1 sequencing

Pooled purified RT-PCR products were used as template to generate eight overlapping amplicons covering PR and RT and 3 amplicons covering codons 51 to 215 in IN. Each codon was interrogated by at least 2 independent amplicons, which were generated in triplicate during 20 cycles of PCR amplification (Platinum® Taq DNA Polymerase High Fidelity, Invitrogen, Carlsbad, CA) followed by pooling and purification of triplicate PCR products (QIAquick PCR Purification Kit, QIAGEN, Valencia, CA). PCR reactions were pooled and purified using the Agencourt AMPure Kit (Beckman Coulter, Benried, Germany) to eliminate primer-dimers. The number of molecules was quantified by fluorometry (Quant-iT PicoGreen dsDNA assay kit, Invitrogen, Carlsbad, CA). When amplicon concentrations were below 5 ng/mL, amplicon size and primer-dimer content were analysed by spectrometry using BioAnalyzer (Agilent Technologies Inc., Santa Clara, CA). Emulsion PCR was carried out as in [17]. Amplicon Sequencing was performed at 454 Life Sciences, Branford, Connecticut, USA, using a Genome Sequencer FLX. Sequences were extracted and aligned against a consensus sequence from all the reads for each sequences region. Clonal sequences resulting with <90% similarity with the consensus sequence were discarded. Sequencing errors in homopolymeric regions were manually inspected and corrected. Sequences that included a stop codon were removed. Unique sequences (haplotypes) were identified and quantified. The frequency of mutants with a Stanford HIV Database [18] score > 5 was determined.

Using a conservative approach, only mutations present in ≥ 1% of the virus population where considered in this study. An in-house analysis of 992 pNL43 clonal sequences obtained by deep sequencing under the same PCR conditions used to generate patient samples showed a mean (SD) nucleotide mismatch rate of 0.07% (0.13%), which is almost identical to previous reports [14] and corresponds to a variability rate of 1.69×10^-5^, within the range of the expected PCR error. The mean (SD) error rate for any aminoacid mismatch was 0.14% (0.19%), but the mean (SD) error rate for actual drug resistance mutations was 0.03% (0.07%). The 99^th^ percentile of mismatches would establish the threshold for nucleotide errors in 0.61% and the limit for identifying true drug resistance mutations in 0.20%.

### Assessment of discrepancies between population and deep sequencing

Genotypes were interpreted with the HIVdb v6.0.8 [18], Rega v8.0.2 [19] and ANRS v18 [20] algorithms. Only mutations with a score ≥ 5 in the Stanford HIV Drug Resistance Database were included in the analysis. Viral susceptibility to each drug was classified as “Sensitive”, “Intermediate” or “Resistant”. Sixty-three paired drug susceptibility predictions (each pair including population and deep sequencing genotypic data) were evaluated per subject and timepoint, 20 for ANRS, 21 for HIVdb v6.0.8 and 22 for Rega v8.0.2.

## Results

### Subjects

The study included 7 individuals, 2 of them women, with previous exposure to a median of 15 antiretrovirals during a median of 13 years. Their median (interquartilic range, IQR) age was 44 (39; 49) years. The median (IQR) year of HIV diagnosis was 1988 (1985; 1994). Median (IQR) HIV-1 RNA levels and CD4 counts were, respectively, 110,600 (46,550; 370,000) copies/mL and 50 (12; 126) cells/mm^3^ at baseline; and 36,700 (2,895; 205,000) copies/mL and 150 (27; 247) cells/mm^3^ at virological failure. Median (IQR) nadir CD4 counts were 14 (1; 152) cells/mm^3^. Deep salvage therapy included darunavir, tipranavir, etravirine or raltegravir in 4, 2, 2 and 5 subjects, respectively. (Table S1) Treatment adherence was adequate in 4 individuals (Subjects 1 to 4) (Table S1) and partial in 2 (Subjects 5 and 6), one individual (Subject 7) (Table S1) interrupted antiretroviral therapy during follow-up.

### Phenotypic susceptibility predictions by population and deep genotyping

The median (IQR) deep sequencing coverage per base was 3938 (3130 – 6945) sequences at baseline and 3965 (3688 – 7091) sequences at virological failure. Deep sequencing detected all mutations found by population sequencing in all subjects, and found additional mutations in 6 out of 7 individuals. (Table S1) Additional mutations were congruent with the treatment history and modified 5.2% of phenotypic susceptibility predictions overall (Table S2), with no significant differences between interpretation algorithms.

Deep sequencing provided limited additional genotypic information at baseline relative to PS. Baseline DS changed the ANRS predicted susceptibility of lopinavir/r and saquinavir/r from sensitive to intermediate in subject 1 and the ANRS susceptibility of darunavir/r from intermediate to resistant in subject 2 (Table S1). In subject 7 (Table S1), however, the detection of minority protease I54L and V82A mutants by deep sequencing in addition to the I54I/V, T74S and L90M detected by population sequencing, consistently decreased the virus susceptibility to indinavir/r across the three algorithms, and led to decreases in predicted susceptibility to saquinavir/r and darunavir/r and to lopinavir/r by the HIVdb and Rega algorithms. The additional detection of the reverse transcriptase M41L and T215Y mutants in this individual led to decreases in susceptibility to different NRTIs by the three algorithms.

Most additional resistance mutations leading to changes in susceptibility predictions were found after virological failure to deep salvage therapy (Table S1). Consistent decreases in etravirine susceptibility were observed in the two subjects treated with etravirine in this study (subjects 1 and 2). Detection of additional mutations in protease in these two individuals also led to decreased susceptibility to saquinavir/r and lopinavir/r in the ANRS algorithm, respectively. Low-level K103N mutants were detected at virological failure in subject 5. This individual had received efavirenz in the past and was receiving nevirapine at baseline, when he was switched from tenofovir, stavudine, lamivudine and nevirapine, to tenofovir/emtricitabine, darunavir/r and enfuvirtide. The withdrawal of stavudine-mediated pressure over pre-existing K103N mutants might have enabled their emergence and subsequent detection at virological failure. Finally, the detection of minority K219Q and T215Y mutants in subjects 6 and 7, respectively, led to consistent decreases in predicted viral susceptibility to different NRTIs.

Most baseline resistance mutations were lost in subject 7 during treatment interruption becoming undetectable even by deep sequencing at the time of virological failure. No changes in predicted susceptibility were observed for tipranavir or raltegravir, although detection of minority G140S in subject 3 suggested improved fitness of the Q148R mutants detected by population sequencing at virological failure (Table S1).

## Discussion

While being technically non-inferior to population genotyping, deep sequencing enabled the detection of additional resistance mutations with potential clinical significance in 6 out of 7 individuals included in this study. Additional mutations, however, only modified about 5% of antiretroviral susceptibility predictions, including decreased etravirine efficacy in the 2 subjects developing virological failure to this drug, and decreased NRTI, efavirenz and indinavir/r efficacy in 2, 1, and 1 subject, respectively. Changes in darunavir susceptibility observed in 2 individuals were not consistent across algorithms. Deep sequencing had no impact on susceptibility predictions for tipranavir or raltegravir.

Deep sequencing could modify patient clinical management by avoiding drugs whose resistance may have been underestimated by population sequencing. Indeed, baseline low-frequency PI-resistant mutants were selected during treatment exposure in two subjects: I54T in subject 1, and V32I, Q58E and L89V in subject 2. I54T is a PI-related mutation that appears to be associated with decreased susceptibility to each of the PIs, although its effect has not been well studied. Q58E is a non-polymorphic PI-related mutation associated with reduced susceptibility tipranavir [21], [22], [23] and possibly to several other PIs. Selection of I54T and Q58E mutations during darunavir therapy may be explained due to residual phenotypic or compensatory effect of such mutations on darunavir susceptibility, genome colinearity with other darunavir-associated mutations, or simple stochastic effects. Interestingly, emergence of I54T in subject 1 was associated with decay in I54A, indicating a possible fitness advantage of I54T over I54A in this particular context. Conversely, substitutions V32I and L89V are nonpolymorphic PI-selected accessory mutations which emerge during treatment with darunavir/ritonavir. Both mutations were associated with decreased response to darunavir/ritonavir in the POWER trials [24], suggesting that they were selected in subject 2 because they conferred additional resistance to darunavir.

Most additional mutants in our study, however, were detected at virological failure. Varghese et al. also detected additional minority variants with major etravirine mutations only in patients failing an NNRTI-containing regimen [14]. Taken together, these results suggest that deep sequencing might be more useful to assess loss of antiviral efficacy after virological failure than to screen for pre-existing resistance in subjects with extensive treatment exposure. As with population sequencing, deep sequencing should also be performed immediately or shortly after virological failure.

The increased sensitivity of deep sequencing could also reassure clinicians about the absence of additional genotypic resistance when making clinical management decisions. This is particularly important for the management of subjects with suboptimal adherence. In our study, deep sequencing provided additional genotypic information in all adherence strata. (Table S1, Table S2) Deep sequencing, for example, confirmed virus susceptibility to integrase strand-transfer inhibitors in 4 out of 5 subjects who developed virological failure to raltegravir-including regimens, indicating that raltegravir remained a suitable option for subsequent salvage regimens. Similarly, deep sequencing confirmed that subjects 5, 6 and 7 could still be treated with a number of different PIs and suggested higher virus susceptibility to tipranavir than to darunavir in subject 7. (Table S2) The absence of resistance mutations by deep sequencing, however, should be interpreted with caution; the loss of most baseline mutants in subject 7 during antiretroviral therapy interruption shows that deep sequencing can also miss clinically relevant resistance mutations present <1% in the viral population.

Detection of low-frequency resistance mutations associated with hypersusceptibility could also improve predicted susceptibility to certain antiretrovirals. For example, the incorporation of a minority T215F mutation in subject 2 changed the HIVdb predicted susceptibility to tenofovir from “resistant” to “intermediate” at virological failure. Therefore, ultrasensitive genotyping also has the potential to refine genotype interpretation rules towards increased virus susceptibility.

Our findings extend those of Le et al. [15], who showed that deep sequencing detected low-frequency mutations unrecognized by Sanger sequencing in 19 out of 22 antiretroviral–experienced individuals experiencing virological failure in routine HIV care between 2004-2007. Additional minority mutants increased a subject's genotypic resistance to one or more antiretrovirals in 17 of 22 individuals (77%), correlated with the failing drugs in 21% subjects, and with historical antiretroviral use in 79% subjects. In Le's study, however, samples were collected before etravirine, darunavir or raltegravir became available, so the effect additional minority mutants on HIV susceptibility to these drugs could not be evaluated.

The main limitations of our study are its small sample size, its retrospective observational nature and the fact that adherence was self-reported. This study makes two arguable assumptions: First, that a mutant detected above 1% is clinically relevant. This threshold is clearly above the PCR and 454 sequencing error found in our own and other studies [14], [25] and has been shown to predict virological outcomes of first-line NNRTI therapy in treatment–naïve individuals.[2] Moreover, potential PCR-derived recombination should not affect our findings because we did not evaluate mutational linkage. The second assumption is that minority mutants have the same weight in genotypic susceptibility interpretation algorithms as if they were present at higher levels. While biologically plausible, formal studies have not been developed to assess this assumption.

In conclusion, in this subset of heavily pre-treated individuals, deep sequencing showed technical non-inferiority to population sequencing. However, although additional mutations improved the assessment of genotypic resistance to etravirine, deep sequencing did not consistently provide additional information on darunavir, tipranavir or raltegravir susceptibility relative to population sequencing. Further studies should extend our findings and address the clinical impact of ultrasensitive genotyping in HIV-1 infected individuals experiencing virological failure to their first or second-line antiretroviral therapy. Proper throughput escalation, sample multiplexing and adequate sequence coverage may potentially turn ultrasensitive genotyping into a feasible strategy for HIV drug resistance management in the clinical setting.

## Supporting Information

Table S1 Additional genotypic information provided by deep HIV-1 sequencing and changes in predicted phenotypic susceptibility, relative to population sequencing ^a, b, c^. ^a^ Only mutations with a score ≥ 5 in the Stanford HIV Drug Resistance Database were included in the analysis. ^b^ ART: antiretroviral therapy; DS: deep sequencing; PI: protease inhibitor; NRTI: nucleoside reverse transcriptase inhibitor; NNRTI: non-nucleoside reverse transcriptase inhibitor; InSTI: Integrase Strand Transfer Inhibitor; ZDV: zidovudine; ABC: abacavir; d4T: stavudine; ddI: didanosine; TDF: tenofovir; EFV: efavirenz; NVP: nevirapine; ETR: etravirine; ATV: atazanavir; IDVr: indinavir/ritonavir; FAPVr: fosamprenavir/ritonavir; SQVr: saquinavir/ritonavir; LPVr: lopinavir/ritonavir; DRVr: darunavir/ritonavir; TPVr: tipranavir/ritonavir; ANRS: algorithm of the French ANRS (*Agence Nationale de Recherche sur le SIDA*) AC11 Resistance group, version 18, July 2009, France; HIVdb: HIV db program version 6.0.8, implemented at the Stanford HIV Drug Resistance Database, Stanford University, USA; REGA: Algorithm of the Rega Institute version 8.0.2, University of Leuven, Belgium. ^c^ Predicted antiretroviral susceptibility: S: susceptible; I: intermediate; R: Resistant.
(DOC)

Table S2 Predicted antiretroviral susceptibility according to Sanger or quantitative deep 454 sequencing. Color code: Green =  Sensitive; Yellow = Intermediate; Red = Resistant. Changes in predicted phenotypic susceptibility between Sanger and 454 sequencing sequencing are highlighted in black boxes. DLV: delavirdine; EFV: efavirenz; ETR: etravirine; NVP: neviapine; 3TC: lamivudine; ABC: abacavir; AZT: zidovudine; d4T: stavudine; ddI: didanosine; FTC: emtricitabine; TDF: tenofovir dippivoxil fumarate; ATV: atazanavir; ATVr: ritonavir-boosted atazanavir; DRVr: ritonavir-boosted darunavir; FPVr: ritonavir-boosted fosamprenavir; IDVr: ritonavir-boosted indinavir; LPVr: ritonavir-boosted lopinavir; NFV: nelfinavir; SQVr: ritonavir-boosted saquinavir; TPVr: ritonavir-boosted tipranavir; RA: ralegravirL; ELV: elvitegravir.
(XLS)
