# Supplementary material for: Added Value of Deep Sequencing Relative to Population Sequencing in Heavily Pre-Treated HIV-1-Infected Subjects
Source: PLoS One. 2011 May 13;6(5):e19461. doi: 10.1371/journal.pone.0019461 (PMC3094345; doi:10.1371/journal.pone.0019461)
Supplement: Table S1 — Additional genotypic information provided by deep HIV-1 sequencing and changes in predicted phenotypic susceptibility, relative to population sequencing a, b, c. a Only mutations with a score ≥ 5 in the Stanford HIV Drug Resistance Database were included in the analysis. b ART: antiretroviral therapy; DS: deep sequencing; PI: protease inhibitor; NRTI: nucleoside reverse transcriptase inhibitor; NNRTI: non-nucleoside reverse transcriptase inhibitor; InSTI: Integrase Strand Transfer Inhibitor; ZDV: zidovudine; ABC: abacavir; d4T: stavudine; ddI: didanosine; TDF: tenofovir; EFV: efavirenz; NVP: nevirapine; ETR: etravirine; ATV: atazanavir; IDVr: indinavir/ritonavir; FAPVr: fosamprenavir/ritonavir; SQVr: saquinavir/ritonavir; LPVr: lopinavir/ritonavir; DRVr: darunavir/ritonavir; TPVr: tipranavir/ritonavir; ANRS: algorithm of the French ANRS (Agence Nationale de Recherche sur le SIDA) AC11 Resistance group, version 18, July 2009, France; HIVdb: HIV db program version 6.0.8, implemented at the Stanford HIV Drug Resistance Database, Stanford University, USA; REGA: Algorithm of the Rega Institute version 8.0.2, University of Leuven, Belgium. c Predicted antiretroviral susceptibility: S: susceptible; I: intermediate; R: Resistant. (DOC) [file pone.0019461.s001.doc]

| **SUBJECT** | **ADHERENCE** | **ART** | **BASELINE** | | | | | **VIROLOGICAL FAILURE** | | | | |
| --- | --- | --- | --- | --- | --- | --- | --- | --- | --- | --- | --- | --- |
|  |  |  | **Mutations by population sequencing** | **Additional mutations by DS (percent in the viral population)** | **Change in Predicted Phenotype due to additional DS data** | | | **Mutations by population sequencing** | **Additional mutations by DS (percent in the viral population)** | **Change in Predicted Phenotype due to additional DS data** | | |
|  |  |  |  |  | **HIVdb** | **REGA** | **ANRS** |  |  | **HIVdb** | **REGA** | **ANRS** |
| 1 | ADEQUATE | TDF FTC DRVr ETR RAL | PI: V11I, V32I, L33F, K43T, I54A, Q58E, T74P, I84V, L89V, L90M | PI: I54T (12.0), V82F (1.1), V82L (1.1) | _ | _ | LPVr, SQVr:  S to I | PR: V11I, V32I, L33F, K43T, I54T, Q58E, T74P, I84V, L89V, L90M | PR: I54A (6.1), V82F (3.5), V82L (3.5) | _ | _ | SQVr:  S to I |
|  |  |  | NRTI: M41L, E44D, D67G, L74IV, V75AITV, V118I, L210W, T215Y, K219N | NRTI: none | _ | _ | _ | NRTI: M41L, E44D, D67G, L74IV, V75T, V118I, L210CW, T215Y, K219DN | NRTI: none | _ | _ | _ |
|  |  |  | NNRTI: L100I, K103N | NNRTI: none | _ | _ | _ | NNRTI: L100I, K103N | NNRTI: V179D (3.0), Y181C (17.6) | ETR:  I to R | ETR:  I to R | ETR:  S to I |
|  |  |  | InSTI: none | InSTI: none | _ | _ | _ | InSTI: none | InSTI: V151I (1.8) | _ | _ | _ |
| 2 | ADEQUATE | TDF FTC DRVr ETR RAL | PI: L33F, K43T, M46L, I54V, T74P, V82A, I84V, L90M | PI: V32I (1.6), F53L (1.6), Q58E (8.6), L89V (21.0) | _ | _ | DRVr:  I to R | PI: V11I, V32I, L33F, K43T, I54A, Q58E, T74P, I84V, L89V, L90M | PI: I54T (15.9) | _ | _ | LPVr:  I to R |
|  |  |  | NRTI: D67N, T69Li, K70R, Y115F, F116Y, Q151M, M184V, T215V, K219Q | NRTI: T215F (1.8) | _ | _ | _ | NRTI: M41L, E44D, D67N, L74V, V75T, V118I, Q151M, M184V, L210W, T215Y, K219N | NRTI: T215F (1.2) | TDF:  R to I | _ | _ |
|  |  |  | NNRTI: K101Q, K103N | NNRTI: none | _ | _ | _ | NNRTI: L100I, K103N | NNRTI: K101E (2.3), Y181C (1.7) | ETR:  I to R | ETR:  I to R | ETR:  S to I |
|  |  |  | InSTI: G163K | InSTI: none | _ | _ | _ | InSTI: none | InSTI: V151I (2.3), G163K (21.5) | _ | _ | _ |
| 3 | ADEQUATE | TDF TPVr RAL | PI: V32I, I54M, Q58E, G73S, I84V, L89V, L90M | PI: none | _ | _ | _ | PI: V32I, I54M, Q58E, G73S, I84V, L89V, L90M | PI: none | _ | _ | _ |
|  |  |  | NRTI: K70KN, M184V, T215Y | NRTI: none | _ | _ | _ | NRTI: K70KN, M184V, T215Y | NRTI: D67N (8.1) | TDF:  S to I | ABC, d4T, ddI:  S to I | _ |
|  |  |  | NNRTI: A98G | NNRTI: none | _ | _ | _ | NNRTI: A98G | NNRTI: none | _ | _ | _ |
|  |  |  | InSTI: none | InSTI: none | _ | _ | _ | InSTI: Q148QR, N155NH, G163GR | InSTI: E138K (32.6), G140S (16.1) | _ | _ | _ |
| 4 | ADEQUATE | TDF FTC TPVr RAL | PI: M46IL, I54V, G73S, I84V, L90M | PI: none | _ | _ | _ | PI: E35G, M46L, I54V, G73S, I84V, L90M | PI: none | _ | _ | _ |
|  |  |  | NRTI: M41L, D67N, L74V, V118I, M184V, L210W, T215Y, K219DN | NRTI: none |  |  |  | NRTI: M41L, D67N, L74V, V118I, M184V, L210W, T215Y, K219N | NRTI: none | _ | _ | _ |
|  |  |  | NNRTI: K101P, V179D, G190A | NNRTI: none | _ | _ | _ | NNRTI: K101P, V179D, G190A | NNRTI: none | _ | _ | _ |
|  |  |  | InSTI: none | InSTI: none | _ | _ | _ | InSTI: none | InSTI: none | _ | _ | _ |
| 5 | PARTIAL | DRVr TDF FTC T20 | PI: L23I, L33F, E35G, I54L, Q58E, N88S | PI: none | _ | _ | _ | PI: L23I, L33F, E35G, I54L, Q58E, N88S | PI: none | _ | _ | _ |
|  |  |  | NRTI: D67N, K70R, M184V, T215Y, K219E | NRTI: T215F (2.2) | _ | _ | _ | NRTI: D67N, K70R, T215Y, K219E | NRTI: T215F (1.7) | _ | _ | _ |
|  |  |  | NNRTI: E138A, Y181V | NNRTI: none | _ | _ | _ | NNRTI: E138A, Y181V | NNRTI: K103N (12.9) | EFV:  I to R | _ | EFV, NVP:  S to R |
|  |  |  | InSTI: E157Q | InSTI: none | _ | _ | _ | InSTI: E157Q | InSTI: none | _ | _ | _ |
| 6 | PARTIAL | ZDV 3TC ABC ATV RAL | PI: None | PI: none | _ | _ | _ | PI: none | PI: none | _ | _ | _ |
|  |  |  | NRTI: D67N, T69DN, K70KR, K219KQ | NRTI: none | _ | _ | _ | NRTI: D67N, T69DN, K70KR, | NRTI: K219Q (1.6) | _ | D4T, ZDV:  I to R  ABC:  S to I | D4T, ZDV:  S to R |
|  |  |  | NNRTI: K103KN | NNRTI: none | _ | _ | _ | NNRTI: K103KN | NNRTI: none | _ | _ | _ |
|  |  |  | InSTI: none | InSTI: none | _ | _ | _ | InSTI: none | InSTI: none | _ | _ | _ |
| 7 | ART INTERRUPTION | STARTS AND STOPS TDF FTC DRVr | PI: I54IV, T74S, L90M | PI: I54L (2.4), V82A (13.9) | IDVr, SQVr:  I to R  DRVr:  S to I | IDVr:  I to R  LPVr:  S to I | IDVr:  I to R | PI: L90M | PI: none | _ | _ | _ |
|  |  |  | NRTI: T215ST | NRTI : M41L (1.4), T215Y (13.7) | ABC, ddI, TDF :  S to I | ddI:  S to I | D4T, ZDV:  I to R  ddI:  S to R | NRTI: none | NRTI: T215Y (7.9) | d4T, ZDV, ABC, ddI, TDF:  S to I | d4T, ZDV:  S to I | d4T, ZDV:  S to R |
|  |  |  | NNRTI: none | NNRTI : none | _ | _ | _ | NNRTI: none | NNRTI: none | _ | _ | _ |
|  |  |  | InSTI: none | InSTI: none | _ | _ | _ | InSTI: none | InSTI: none | _ | _ | _ |
